# Altered expression of podoplanin in keratocystic odontogenic tumours following decompression

**DOI:** 10.3892/ol.2013.1764

**Published:** 2013-12-17

**Authors:** XIAOMIN ZHANG, JING WANG, XU DING, SHUZHONG XING, WEI ZHANG, LIZHEN WANG, HEMING WU, LIN WANG

**Affiliations:** 1 Institute of Stomatology, Nanjing Medical University, Nanjing, Jiangsu 210029, P.R. China; 2 Division of Oral Pathology, The Shanghai Ninth People’s Hospital, Shanghai 200011, P.R. China

**Keywords:** keratocystic odontogenic tumour, decompression, podoplanin, immunohistochemistry

## Abstract

Marsupialisation or decompression is frequently performed as a conservative therapy for keratocystic odontogenic tumours (KCOTs). Positive podoplanin (PDPN) expression in the epithelium of KCOT has been previously reported and may be associated with neoplastic invasion. In the present study, changes in PDPN expression were observed in the epithelium of KCOTs following decompression. In total, 16 pairs of paraffin-embedded tissue specimens obtained at the time of decompression and at two-stage curettage or enucleation were collected and immunohistochemically examined using an antibody against PDPN. The intensity of PDPN staining was evaluated with a semi-quantitative detection method and statistically analysed. The immunohistochemical reactivity of PDPN was consistently markedly positive in 93.8% of KCOT samples prior to decompression. The positive staining was immunolocalised to the cell membrane and cytoplasm of cells in the basal layer and extended into the suprabasal layer for two to three cell layers. At the time of curettage, 2 of the 16 (12.5%) cases were completely negative, 11 of the 16 (68.8%) cases were locally positive and 3 of the 16 (18.7%) cases showed a ‘linear staining’ pattern, as the PDPN-positive cells were restricted to within the single basal layer. The expression level of PDPN was significantly decreased (P<0.05) and a significant loss or reduction of PDPN expression was observed in KCOTs following decompression. Larger sample groups are required to further verify this result.

## Introduction

According to the 2005 World Health Organization classification, keratocystic odontogenic tumour (KCOT), which was previously termed odontogenic keratocyst, is categorised as a benign odontogenic tumour (1). Several factors justified this decision (2), including the following: i) Clinically, KCOT behaves as a locally destructive and highly recurrent lesion; ii) histopathologically, it is typical to observe the basal epithelial layer budding into the connective tissue and the absence of mitotic figures in the suprabasal layer; and iii) genetically, mutations of the tumour suppressor gene, Patched 1, in systemic or sporadic KCOT were successively reported by Lench *et al* (3) and Barreto *et al* (4), indicating that dysregulation of the Hedgehog signalling pathway is involved in the molecular pathogenesis.

At present, marsupialisation or decompression combined with two-stage curettage or enucleation is a common treatment for large KCOT. This treatment has the advantage of minimal invasiveness and the preservation of appearance and function (1). This procedure decreases the size of the lesion (5) and in specific circumstances, leads to a lower recurrence rate (1), although a final conclusion has not been reached with regard to this potential benefit (2). To improve the outcome of decompression, it is necessary to elucidate the underlying molecular mechanisms of this therapy.

Podoplanin (PDPN), a 36–43-kDa mucin-like transmembrane glycoprotein, is widely used as a marker for lymphatic endothelial cells (6). However, recent studies have shown that the protein is also expressed in a variety of normal tissues, as well as neoplastic tissues in pathological and physiological settings (6,7). This molecule has diverse functions, including regulation of organ development, cell motility, tumourigenesis and metastasis (6,8,9). Previous studies (7,10) have reported that PDPN is markedly expressed in KCOTs, as in the cases of ameloblastoma and adenomatoid odontogenic and calcifying cystic odontogenic tumours, but negative in orthokeratinised odontogenic cysts (OOCs) and dentigerous cysts, suggestive of its neoplastic nature. It is likely that the upregulated expression of PDPN has a role in the processes of tumour proliferation and invasion.

Following decompression, the intracystic environment of KCOTs change. Furthermore, several previous studies found that the typical histological features were lost (11,12) and that a variety of biomarkers associated with proliferation (12), invasion, cell differentiation (13) or apoptosis (14) were downregulated following the therapy. To the best of our knowledge, the manner in which the expression of PDPN changes following decompression has not been previously reported. Through the immunochemical detection of 16 cases, the present study is the first to report that the PDPN expression in KCOTs is notably downregulated following this therapy.

## Materials and methods

### Patients

The presents study analysed the cases of 16 patients with histologically confirmed KCOTs from the Stomatology Hospital of Jiangsu Province (Nanjing, China) and the Shanghai Ninth People’s Hospital (Shanghai, China). Recurrent cases or those associated with nevoid basal cell carcinoma syndrome were excluded. All patients underwent decompression surgery followed by two-stage cyst enucleation. The clinical information of the patients is shown in Table I. Post-operative follow-up was comprised of clinical and radiographic examinations between January 2004 and September 2012. The average duration of draining and irrigation prior to the two-stage surgery was 19.5 months (range, 6.5–44.0 months).

Paraffin specimens of the tissue samples that were obtained at the time of decompression and enucleation were gathered from the Division of Oral Pathology of the Stomatology Hospital of Jiangsu Province and the Shanghai Ninth People’s Hospital. All patients provided signed informed consent prior to enrollment and the study was approved by the Ethics Committees of the Shanghai Ninth People’s Hospital and the Jiangsu Stomatological Hospital.

### Immunohistochemical evaluation

Each of the 16 pairs of formalin-fixed, paraffin-embedded samples were sectioned continuously into two 4-μm slices and mounted onto glass microscope slides. One slice of each pair was prepared for immunohistochemical (PDPN) analysis and the other was used as a negative control by substituting phosphate-buffered saline for the anti-PDPN as a primary antibody. Positive staining for PDPN at the lymphatic vessels in connective tissues was used as the positive control.

Briefly, the deparaffinised sections were immersed in absolute methanol containing 0.3% H_2_O_2_ for 15 min at room temperature to block endogenous peroxidase activity. Following washing, the sections were immersed in 0.01 M citrate buffer (pH 6.0) and heated in a microwave oven at 95°C for 5 min. A properly diluted (1:50) rabbit monoclonal anti-human D2–40 (PDPN) antibody (Proteintech Group, Inc., Chicago, IL, USA) was then applied to the sections at 4°C overnight, followed by a prediluted anti-mouse IgG antibody conjugated with peroxidase (Nichirei Corporation, Tokyo, Japan) for 1 h at room temperature. The sections were immersed for 8 min in 0.05% 3,3′-diaminobenzidine tetrahydrochloride in 0.05 M Tris-HCl buffer (pH 8.5) containing 0.01% H_2_O_2_ and then counterstained with haematoxylin.

### Criterion for immunohistochemical evaluation

The immunohistochemical reactivity for PDPN was evaluated using a semi-quantitative detection method, which is referred to in the previous literature (10), and classified into three groups according to the following intensity scores: 0, negative (−); 1, weakly to moderately positive (+); and 2, strongly positive (++). The evaluation was performed by two experienced pathologists who were each blinded to the clinical information provided.

### Statistical analysis

Student’s paired t-test was used to evaluate the altered immunoreactivity of PDPN in KCOTs at the time of enucleation compared with that at the time of decompression. SPSS 17.0 software (SPSS, Inc., Chicago, IL, USA) was used for statistical analysis. Specific data are shown in Table I and P<0.05 was considered to indicate a statistically significant difference.

## Results

### Immunohistochemistry analysis

In the present study, patients with samples obtained at the time of decompression were assigned to group I and those with samples obtained at the time of two-stage enucleation were assigned to group II.

**Figure 1:**
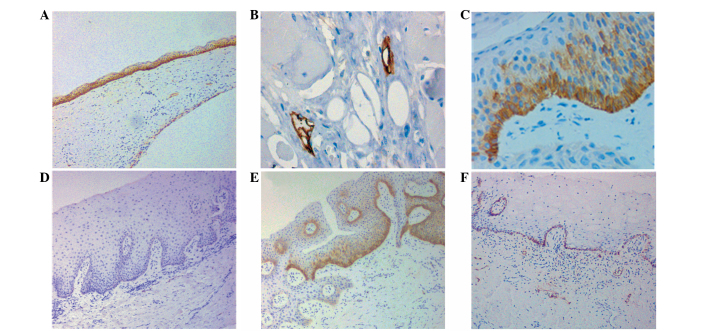
PDPN expression levels in KCOT epithelium. (A) Section of PDPN staining in the cystic lining of KCOT prior to decompression (magnification, ×100). (B) Strong positive staining for PDPN in lymphatic endothelial cells as a positive control (magnification, ×400). (C) Section of PDPN staining in the cystic lining of KCOT prior to decompression (magnification, ×400). (D) Entirely negative, (E) locally positive and (F) linearly positive staining for PDPN in the cystic lining of KCOT following decompression (magnification, ×100). KCOT, keratocystic odontogenic tumour; PDPN, podoplanin.

The immunohistochemical staining pattern of PDPN in KCOTs was membranous and cytoplasmic (Fig. 1A). In group I, 15 of the 16 cases (93.8%) showed strong PDPN expression in the epithelium, as with the expression of the lymphatic endothelial cells in the positive control (Fig. 1B), while 1 of the 16 (6.2%) cases was negative. All PDPN-positive cells were restricted to the basal layer of the epithelial lining, with a small amount of extension (2–3 layers of cells) toward the parabasal or lower middle layers; the upper layers were negative for this protein (Fig. 1C).

In group II, 3 of the 16 cases showed strong or negative PDPN expression, each consistent with its corresponding counterpart in group I. In the remaining cases, PDPN expression was significantly decreased; one case was entirely negative in all visual fields (Fig. 1D), 7 cases showed localized positive staining for PDPN, indicating that the segmental PDPN-positive areas were only found in sporadic regions and coexisted with the PDPN-negative areas (Fig. 1E) and in 4 cases, the PDPN-positive cells were restricted to a single layer (the basal layer), while the adjacent prickle-cell layer was negative (Fig. 1F).

### Intensity scoring

The mean intensity score in group I was 1.87 (0.27), while the mean intensity score in group II was 1.00 (0.29). The P-value of group I versus group II in PDPN staining intensity was 0.0001. The statistical analysis showed a significant decrease in the expression of PDPN in group II compared with that in group I.

## Discussion

Marsupialisation or decompression is frequently used as a conservative therapy for large KCOTs. It has been previously reported that tumour size is evidently decreased following decompression (5). For example, Nakamura *et al* (12) reported that 96% of cases showed a cystic reduction of ≥50%. Histologically, the typical features of KCOTs were lost following decompression and took the form of hyperplastic epithelium, thickened fibrous lamina and increased inflammatory infiltration (11,12). Similarly, in the present study, the histological features of 13 samples in group II changed in the same way. In the field of molecular biology, a series of biomarkers highly expressed in KCOTs, including IL-1α-induced prostaglandins, collagenase (15), Ki-67 (12,15), bcl-2 (14) and keratinocyte growth factor (16), have been reported to notably decrease, indicating the attenuation of cell proliferation, antiapoptosis and local invasion.

PDPN is a glycoprotein involved in the rearrangement of the cytoskeleton. A number of previous *in vitro* and *in vivo* studies have shown that PDPN is involved in cell motility and tumour invasion and metastasis (8). In addition, PDPN reportedly promotes the formation of elongated cellular extension and increased adhesion and cell migration. As a regulator of cell morphology, PDPN exhibits actin remodelling properties, including the prevention of stress fibre and filopod formation (17).

The positive expression of PDPN in KCOTs may reflect neoplastic activity or cell proliferation and invasion. A study by Caetano *et al* (18) showed a statistically significant correlation between PDPN expression and the cellular proliferation index (Ki-67) of KCOT and OOC. The study revealed that KCOTs demonstrated a higher proliferative activity accompanied by stronger PDPN expression, while in its indolent counterpart, OOC, the Ki-67 index and PDPN expression intensities were lower. Similarly, Tsuneki *et al* (7) found that in KCOTs, PDPN-positive cells were located within areas of proliferating cell nuclear antigen (PCNA)-positive cells representative of the cell proliferation centre. In addition, the study revealed that PDPN-positive cells were overlapped by cells positive for the extracellular matrix-related molecules, integrin-β1, fibronectin and matrix metalloprotein-9 (MMP-9). Therefore, the authors hypothesised that PDPN is involved in extracellular matrix remodelling and cell growth in odontogenic tumours (7). Notably, Caetano *et al* (18) concluded that PDPN-positive staining was always found in the peripheral regions of tumour cell nests, the basal layers of epithelium and areas of high cellular activity, such as daughter cysts of KCOTs and secreting ameloblasts of ameloblastic fibro-odontomas. Above all, these results indicated that PDPN possibly has a role in the process of tumoural invasion.

To the best of our knowledge, the present study is the first report to compare the altered expression of PDPN in KCOTs between one-stage decompression and two-stage enucleation. In the current study, 93.8% of cases (15/16) showed a markedly positive expression in the epithelium that was restricted to the basal layer and extended into the stratum spinosum for two to three cell layers, consistent with the observations of previous studies. At the time of enucleation, only three cases showed the same level of PDPN expression as that prior to decompression, and in all the remaining samples, PDPN expression was significantly decreased. The evidence that the immunoreactivity of PDPN correlates with a series of proteins involved in cell proliferation and tumourous invasion, including Ki-67 (18), PCNA (7) and MMP-9 (7), further confirms that the process of cell proliferation and the local destruction of KCOTs are attenuated by decompression. However, due to the small sample size of the present study, this conclusion requires further investigation.

The concrete mechanisms of PDPN in the pathogenesis and progression of KCOTs remain unclear. Few previous studies have analysed the correlation between PDPN expression and the clinical behaviour of KCOTs, such as the tendency to recur. In the present study, although a notable change was observed in PDPN expression following decompression, the potential correlation between this change and the clinical features of KCOTs, including the duration of decompression, tumour location and recurrence rate, were not analysed due to the small sample size and short follow-up period. These questions remain to be further investigated.

## Figures and Tables

**Table I:** Patient clinical information and intensity of PDPN expression.

Case	Age, years	Gender	Duration of decompression, min	Location	Outcome (degree of PDPN expression)^a^	

					Group I	Group II
1	22	M	27	Mandible; Mol-Ram	2	1
2	15	M	10	Mandible; Ang-Ram	2	1
3	20	M	21	Mandible; Ang-Ram	2	1
4	42	M	17	Mandible; Mol-Ram	2	1
5	20	F	16	Mandible; Ang-Ram	0	0
6	13	F	12	Mandible; Mol-Ram	2	1
7	38	F	3	Mandible; Ang-Ram	2	1
8	34	F	16	Mandible; Mol-Ram	2	1
9	49	F	18	Mandible; Ang-Ram	2	1
10	55	M	15	Maxilla; Ant	2	1
11	25	F	15	Mandible; Mol-Ram	2	2
12	33	F	9	Mandible; Ang-Ram	2	1
13	35	M	23	Mandible; Ang-Ram	2	0
14	24	F	31	Mandible; Ang-Ram	2	1
15	29	M	23	Maxilla; Ant	2	2
16	28	F	27	Maxilla; Ant	2	1

Group I samples were obtained at the time of decompression and group II samples were obtained at the time of two-stage enucleation.

a Immunohistochemical activity was measured as follows: 2, strongly positive; 1, weakly to moderately positive; and 0, negative. PDPN, podoplanin; M, male; F, female; Ant, anterior region; Ang, angular region; Mol, molar region; Ram, mandibular ramus.
